# Radioprotective Agents and Enhancers Factors. Preventive and Therapeutic Strategies for Oxidative Induced Radiotherapy Damages in Hematological Malignancies

**DOI:** 10.3390/antiox9111116

**Published:** 2020-11-12

**Authors:** Andrea Gaetano Allegra, Federica Mannino, Vanessa Innao, Caterina Musolino, Alessandro Allegra

**Affiliations:** 1 Radiation Oncology Unit, Department of Biomedical, Experimental, and Clinical Sciences “Mario Serio”, Azienda Ospedaliero-Universitaria Careggi, University of Florence, 50100 Florence, Italy; andrea.allegra@hotmail.it; 2 Department of Clinical and Experimental Medicine, University of Messina, c/o AOU Policlinico G. Martino, Via C. Valeria Gazzi, 98125 Messina, Italy; fmannino@unime.it; 3 Department of Human Pathology in Adulthood and Childhood “Gaetano Barresi”, Division of Haematology, University of Messina, 98125 Messina, Italy; vinnao@unime.it (V.I.); cmusolino@unime.it (C.M.)

**Keywords:** radiotherapy, hematological malignancies, oxidative stress, lymphoma, leukemia, multiple myeloma, apoptosis, mitochondria

## Abstract

Radiation therapy plays a critical role in the management of a wide range of hematologic malignancies. It is well known that the post-irradiation damages both in the bone marrow and in other organs are the main causes of post-irradiation morbidity and mortality. Tumor control without producing extensive damage to the surrounding normal cells, through the use of radioprotectors, is of special clinical relevance in radiotherapy. An increasing amount of data is helping to clarify the role of oxidative stress in toxicity and therapy response. Radioprotective agents are substances that moderate the oxidative effects of radiation on healthy normal tissues while preserving the sensitivity to radiation damage in tumor cells. As well as the substances capable of carrying out a protective action against the oxidative damage caused by radiotherapy, other substances have been identified as possible enhancers of the radiotherapy and cytotoxic activity via an oxidative effect. The purpose of this review was to examine the data in the literature on the possible use of old and new substances to increase the efficacy of radiation treatment in hematological diseases and to reduce the harmful effects of the treatment.

## 1. Introduction

### Radiotherapy in Hematologic Malignancies

Radiation therapy (RT) has an essential role in the treatment of a wide range of hematologic malignancies. The ideal radiation dosage and target volume, and efficacious system of combining radiation with systemic drugs, differ in relation to the histologic varieties, stage, and patient performance status. Reducing dosages to adjacent organs without losing disease control is of leading importance as it can increase the therapeutic ratio of subjects getting RT for hematologic malignancies [1].

Lymphomas are very radiosensitive tumors and RT is the principal treatment approach, and it is the most efficacious single therapy for local control of lymphomas. Nevertheless, curative objective is only imaginable if all lymphoma tissue can be included in the zone to be irradiated with the recommended total irradiation dosage. For this reason, RT is a single mode only in early stages of Hodgkin’s lymphoma and low-grade non-Hodgkin’s lymphoma. However, in most subjects, RT can be employed as consolidation treatment after chemotherapy. In the last few years, irradiation treatment of lymphomas has been improved in order to save essential tissues and decrease toxicity. However, although efficacious, RT is a disregarded type of therapy because of the concern of side effects after irradiation [2].

RT is also an efficacious treatment for multiple myeloma (MM) [3]. External beam irradiation is habitually employed for palliation of bone pain and treatment of solitary plasmacytomas [4]. In MM subjects who have diffused bone disease, systemic RT with compounds such as bone-seeking radionuclides [5,6] or treatments such as intensity modulated RT and helical tomography [7,8] have been employed. For instance, bone-seeking radionuclide treatment with radioactive samarium conjugated to a tetraphosphonate chelator has been utilized in myeloablative protocols for MM [9,10,11,12]. Moreover, the combinate use of RT with chemotherapeutic drugs such as bortezomib or thalidomide [13,14,15] has demonstrated good clinical effects with decreased radiotoxicity to normal tissues.

Finally, as far leukemic patients, the most frequent situation in which RT therapy is employed comprises therapy of central nervous system (CNS) disease and other types of extramedullary relapses. Either whole-brain radiation treatment or cranial spinal irradiation may be evaluated in subjects with leukemia and history of CNS disease as part of therapy before allogeneic stem cell transplant (SCT), consolidation after salvage chemo treatment, or salvage therapy. Myeloid sarcoma, also recognized as granulocytic sarcoma, and leukemia cutis are extramedullary malignancies containing myeloid blasts, in most patients arising concomitantly with acute myeloid leukemia, and they are extremely sensitive to RT [16].

## 2. Radiotherapy and Oxidative Stress

An expanding quantity of results is aiding to explain the effects of oxidative stress in malignancies both in the onset of the disease and in response of the treatment. In several tumoral situations, reactive oxygen species (ROS) generation and removal are out of control, leading to disproportionate quantities of ROS [17]. Antioxidants are essential in the removal of ROS, so preserving the normal physiological condition is vital [18].

An oxidatively balanced redox status may influence carcinogenesis by modifying DNA and different cellular components. On the other hand, oxidative stress increases the activities of several cytostatic drugs by provoking sublethal DNA injury and so stimulates programmed cell death. Moreover, oxidative stress markers are prognostically relevant in solid cancers [19,20,21], but also in multiple myeloma, lymphoma, and acute and chronic leukemia [21,22,23,24,25,26,27].

On the other hand, it is well known that, at molecular level, the cytotoxic actions of ionizing radiation (IR) on tumor and normal cells is essentially due to ROS and reactive nitrogen species (RNS) generation such as the hydrogen peroxide, superoxide anion, hydroxyl radical, and nitrogen dioxide via the radiolysis of cellular water [28]. If not scavenged, these ROS/RNS have a main action in the cell injury by provoking lipid peroxidation, DNA strand breaks, and protein modifications. ROS/RNS also induct the onset of different biochemical and molecular signaling effects that may either fix the injury or provoke cell death, such as apoptosis [29,30].

Among the several compounds that participate in regulating the process, the proto-oncogene bcl-2 family plays a main role. B-cell lymphoma 2 (Bcl-2) is an intracellular protein that locates to endoplasmic reticulum, mitochondria, and the nuclear envelope. It blocks programmed cell death and augments cell survival under different situations in various cell types [31,32]. The expression of Bcl-2 is intensely connected with the expression of several antioxidant enzymes and the strong relationship controlling the intracellular redox concentration has an essential action in cell destiny [33].

However, after exposure of cells to RT, the stimulation of caspases is moderately faint, and then the signal is directed via the mitochondria for efficacious death execution (type 2 or intrinsic signaling).

In fact, damage of cellular DNA after RT is a dose-dependent event and happens in both the nuclear (nDNA) and extra-nuclear DNA. Thus, besides nDNA, mitochondrial DNA (mtDNA) is similarly altered [34,35]. Several experimentations reported that mtDNA can be a target for endogenous ROS and free radicals generated by IR [36,37]. The modalities of cellular response to RT with regard to mtDNA modifications are essentially two different mechanisms. Firstly, mtDNA has limited repair systems and mitochondrial function is maintained principally due to its high copy number. One potential radio-protective system is that increased replication of mtDNA decreases the mutation occurrence of total mtDNA and reduces the onset of lethal radiation injury to the mitochondria [38,39]. This hypothesis has been supported by Zhang et al. by demonstrating augmented mtDNA copy number in bone marrow (BM) of total body irradiated animals [40].

As for the second mechanism, IR generally stimulates cell apoptosis by provoking a gathering of large scale mtDNA deletions, particularly the specific 4977 bp deletion, denoted to as the “common deletion (CD)” [41]. The site of CD is bordered by two 13 bp direct repeats (ACCTCCCTCACCA) at mtDNA nucleotide site 8470 and 13,447 [42]. Findings have demonstrated that CD can be a precise indicator of oxidative damage to mtDNA [43,44,45]. Wen et al. studied mtDNA modifications in irradiated human peripheral lymphocytes from acute lymphoblastic leukemia (ALL) subjects [46]. Significant inverse correlation was demonstrated between CD level and mtDNA content. CD content and mtDNA may be believed as prognostic factors to radiation toxicity [46].

However, it is well known that the post-irradiation injuries in the BM and diverse organs represent a main problem and are the principal reasons of post-irradiation mortality and morbidity [47].

The purpose of this review was to examine the data in the literature on the possibility of using old and new substances to increase the efficacy of radiation treatment in hematological diseases and to reduce the harmful effects of the treatment.

## 3. Radioprotective Agents

Cancer management without causing wide injury to the adjacent normal cells, via the employment of radioprotectors, is of exceptional clinical importance in RT. Radioprotective factors are compounds that temper the consequences of radiation on normal tissues, maintaining the sensitivity to radiation injury in tumor cells [29]. However, no ideal radioprotector has been obtainable so far that would be non-toxic at its best, at an efficacious dosage, and that could defend normal cells against radiation damage while maintaining the radiosensitivity of tumor cells.

A fragile equilibrium exists between oxidant and antioxidant mechanisms under physiological situations, but it is compromised in pathologic conditions. Oxidative stress activation during several diseases may reduce the antioxidant ability of cells, and vulnerability of target elements to oxidative stress increases as a result. Enzymatic and nonenzymatic antioxidants avoid surplus ROS generation and counteract the ROS. Substances such as vitamin A, vitamin C, vitamin E, catechin, epicatechin, carotenoids, and in vivo low-molecular weight antioxidants (melatonin, lipoic acid, uric acid, haptaglobuline, bilirubin, and melatonin) represent the total antioxidant capacity of plasma [48].

Among the great number of natural and synthetic radioprotective substances, the phytochemical compounds, comprising phenolics (simple phenols, benzoic acid derivatives, flavonoids, stilbenes, phenylpropanoids, tannins, lignins, and lignans) have provided encouraging effects because of their capacity to scavenge free radicals and reduce toxicity [49] (Table 1). However, the possible use of substances, such as genistein and quercetin, has been impeded due to the inadequate water-solubility, and therefore their reduced bioavailability [50,51].

**Figure 1:**
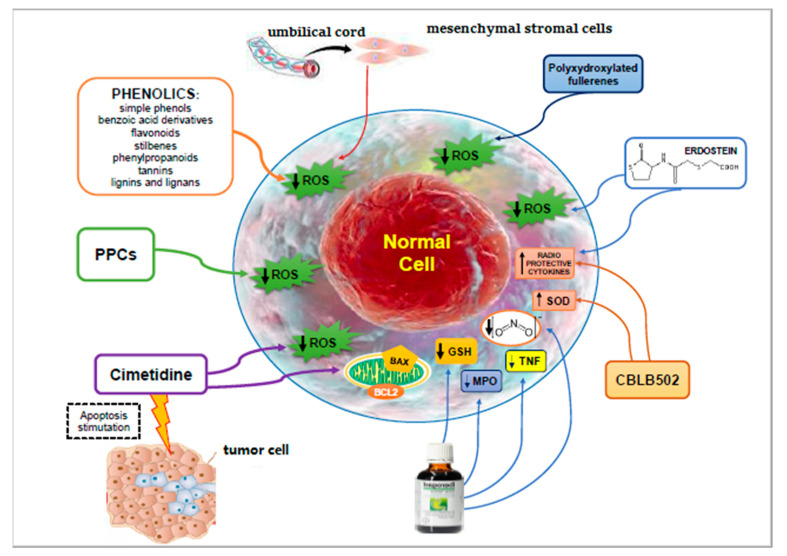
Natural and synthetic radioprotective substances.

The polyphenolic-polysaccharide conjugates (PPCs) extracted from the plants of *Rosaceae* family (*Rubus plicatus*, *Sanguisorba officinalis* L., and *Fragaria vesca* L.) as well as of *Asteraceae* family are compounds for distinguishing the secondary cell wall of higher plants (Figure 1). The PPCs are water-soluble, non-toxic polymeric substances [52]. It was reported that the PPCs were capable of augmenting the post-radiation survival of peripheral blood mononuclear cells (PBMC)s by blocking programmed cell death, while they did not defend the leukemic cells against radiation-induced programmed cell death. The PPCs guarantee an efficacious defense of PBMCs via scavenging of intracellular ROS and reducing DNA injury, while they offered no decrease of the oxidative stress and DNA injury in K562 cells (a human immortalized myelogenous leukemia cell line). These results intensely propose that the PPCs, principally those extracted from *Elodea canadensis* and *S. officinalis*, can selectively defend normal cells, thus they have the benchmarks of radioprotectors for possible employment in RT [53].

A diverse possible radioprotective drug could be Cimetidine, a histamine type II receptors blocker, which has been demonstrated to have an antisuppressor action. Cimetidine has also been employed efficaciously to reestablish immune activities in subjects with malignancies, hypogammaglobulinemia, and acquired immune deficiency syndrome-related complexes [77]. Some in vitro and in vivo experiments evaluated the radioprotective activity of cimetidine on lympho-hematopoietic cells. The mechanism by which cimetidine decreases the mutagenic action of radiation is not well-known, even if a hydroxyl radical scavenging mechanism has been proposed [54]. Estaphan et al. studied the protective action of cimetidine in animals exposed to γ-irradiation and evaluated the Bcl2/Bcl2 associated X (bax) pathway as a possible fundamental mechanism [55]. Cimetidine pretreatment considerably reduced the fibrosis, the loss of BM cell count, and the intestinal lining destruction reported in the untreated irradiated animals, and drastically reduced the oxidative stress, inflammation, and Bax/Bcl2 ratio. An important relationship between Bax/Bcl2 ratio, tissue oxidative stress level, and tissue injury was demonstrated. Cimetidine could be a very encouraging radioprotective drug with a possible differential beneficial action on both cancer cells (stimulating programmed cell death) and contiguous normal cells (provoking radioprotection through blocking programmed cell death) via its antioxidant action and consequent control of type 2 apoptotic pathway [55].

However, Waller Reed Army Research Institute produced and examined over 4000 compounds that could have a radioprotective activity. WR2721 (Amifostine) was the most efficacious substance, although its use is restrained due to its accumulative toxicity [56]. So, there is still a persistent necessity to find new, efficacious, and non-toxic radio-protective substances.

Some compounds seem to be capable to exercise a protective effect on specific consequences of the RT, being able to decrease the damaging actions effects on particular organs or systems such as the intestinal mucosa, the male reproductive system, kidney, heart, or bone marrow.

Organ or system healing can be accomplished ex vivo, by administration of embryonic or adult stem cells, or in situ, by dispensing elements that will stimulate or improve the healing mechanisms of the organ itself, and several efforts have been made about the elaboration of a system for efficacious growth factors release in vivo to promote cellular repair and tissue renewal [78].

As far intestinal mucosa, radiation enteritis is a grave complication caused by the cytotoxic action of RT on the intestinal mucosa. The genesis of this situation implicates the delivery of pro-inflammatory cytokines and ROS and augments programmed cell death in the intestinal epithelium, provoking an alteration of intestinal structure and function [79,80]. Research evaluated the possible radio-protective and anti-apoptotic actions of STW5 (Iberogast^®^,) an herbal preparation, inclosing extracts of *Angelica archangelica*, *Silybum marianum*, *Melissa officinalis*, *Iberis amara*, *Matricaria recutita*, *Carum carvi*, *Mentha piperita*, *Chelidonium majus*, and *Glycyrrhiza glabra* [57].

Exposure to radiation caused apoptotic modifications associated with an augmentation in cytosolic calcium, reduction of complex I, mitochondrial cytochrome c, and B-cell lymphoma-2. Oxidative stress parameters were disturbed, while inflammation markers such as tumor necrosis factor and indicators of intestinal damage such as serum diamine oxidase were augmented. STW 5 defended against histological modifications and stabilized the deranged parameters [58].

The powerful antioxidant action of STW5 was reported in numerous other experimental situations [59] and was demonstrated by the reduction of glutathione(GSH) levels and lessening of the increased thiobarbituric acid reactive substances and nitrite concentrations in the intestinal tissue [60]. STW5 avoided the increase of myeloperoxidase and TNF levels in intestinal tissue caused by RT as well as the increase of serum level of diamine oxidase. The antioxidant and anti-inflammatory actions of STW5 have been ascribed, at least in part, to the flavonoid and phenolic carboxylic acid content of its components [57].

A different interesting compound is CBLB502, a polypeptide originated from *Salmonella* flagellin. This compound connects to the Toll-like receptor 5 (TLR5) and stimulates nuclear factor-kappa B (NFκB), a main controller of programmed cell death, inflammation, and immune response. A single administration of CBLB502 before lethal IR doses was demonstrated to defend animals from gastrointestinal and hematopoietic acute radiation syndrome, but not tumor expansion [61]. CBLB502 administration induces the expression of the powerful natural antioxidant superoxide dismutase (SOD)2 and stimulates the expression of numerous radioprotective cytokines, such as IL-6, G-CSF, and TNF-α [61]. CBLB502 may theoretically increase the therapeutic index of RT and act as a new substance stopping irradiation injury [62]. Moreover, CBLB502 displays immunotherapeutic ability by stimulating TLR5-expressing accessory immune cells [63,64,65].

IR can also cause damage to the male reproductive system in subjects experiencing RT [81,82]. The testis, one of the most radiosensitive organs, can be altered by low IR dosages as of the existence of replicating spermatogonial cells [83]. Abdominal irradiation during RT may cause the accrual of harmful radiation concentrations in the testes. Previous studies demonstrated that IR can alter physiologic spermatogenic metabolism, growth, and differentiation, provoking low sperm counts, sterility, and sexual alterations [84,85,86].

Numerous sorts of antioxidants, such as hydrogen-rich saline, have been studied for the treatment of IR-caused testicular damage, with the intention of increasing the fertility of patients [87,88,89]. CBLB502 was also stated to reestablish testicular antioxidant condition and decrease testicular oxidative injury caused by IR [66]. To evaluate if CBLB502 can reduce testicular damage, animals were intraperitoneally injected with CBLB502 prior to applying IR [67]. It was reported that CBLB502 administration reduced IR-provoked oxidative stress, decreased the alterations of architecture of seminiferous tubules, increased the sperm quality and quantity, and ameliorated male mouse fertility. Moreover, CBLB502 effectively decreased DNA injuries. CBLB502 was reported to stimulate the NFκB pathway and decrease the programmed cell death rate with an augmentation in anti-apoptotic B-cell lymphoma 2 concentrations. Furthermore, an IR-caused decrease in serum testosterone and superoxide dismutase (SOD) concentrations and an augmentation in malondialdehyde (MDA) concentrations were significantly inverted in CBLB502-pretreated animals. No relevant actions were reported in *TLR5* knockout mice, proposing that defense of the testis against IR by CBLB502 is essentially due to the TLR5 signaling pathway [67].

A different side effect of RT is the radiation-caused kidney damage, particularly radiation nephropathy. The frequency of RT-caused kidney injury is probably underrated because of the protracted latency and being often ascribed to diverse causes [90,91]. In RT nephropathy, renal endothelial alterations and modified hemodynamics are well-known elements [92,93].

The radiation-caused damage is principally due to the production of ROS, which causes an imbalance between pro-oxidant and antioxidant elements within the cell and, in turn, oxidation of DNA, lipids, proteins, and cell death [94]. Moreover, there is some proof for programmed cell death as the mechanism of renal tubular cell loss in RT nephropathy [95,96,97].

A homocysteine derivative, Erdosteine, is recognized to have protecting actions on the delivery of free oxygen radicals alongside its mucolytic effect. A study evaluated the potential protective action of Erdosteine against radiation-caused renal alterations in rats [68]. The findings demonstrated that the use of Erdosteine in rats before irradiation ameliorated the altered kidney function. Moreover, IL-1, IL-6, and TNF-alpha circulating levels were also significantly improved. Kidney glutathione peroxidase and catalase levels and reduced glutathione levels displayed almost normal concentrations with respect to the irradiated group.

Erdosteine operates in the kidney as a powerful scavenger of free radicals and might offer relevant protection against RT-caused inflammatory kidney injury [68].

Irradiation myocardial fibrosis (IMF) is a diverse complication correlated with RT for hematopoietic stem cell transplantation, and lymphoma [98]. A research evaluated the paracrine actions of human umbilical cord-derived mesenchymal stromal cells (UC-MSCs) in an experimental model of IMF [69]. Primary human cardiac fibroblasts (HCF) cells were exposed to radiation and cultured with the conditioned medium of UC-MSCs (MSCCM). MSCCM increased cell viability, decreased collagen deposition, inhibited oxidative stress, augmented antioxidant status, and diminished pro-fibrotic IL-6, IL-8, and TGF-β1 concentrations [69].

Furthermore, as regards to bone marrow toxicity, calf spleen extractive injection (CSEI), a small peptide, has been employed to ameliorate leucopenia and thrombocytopenia in subjects who experienced RT [70,71,72].

Polyxydroxylated fullerenes have also been recently established as exogenous redox balance controllers, able to produce anti-oxidative actions [73]. Examinations of biological consequences of fullerenol have offered confirmation for its ROS/RNS scavenger abilities in vitro and radioprotective efficacy in vivo [74].

Finally, FDA authorized Palifermin to decrease the occurrence and duration of grave oral mucositis in subjects with hematological diseases. Palifermin is a recombinant N-terminal truncated form of endogenous keratinocyte growth factor (KGF) with activity analogous to that of the KGF, but with augmented stability. Palifermin stimulates cell proliferation and increases cytoprotective systems. Thus, palifermin may inhibit epithelial cell apoptosis and block injury to the epithelial DNA, decrease the delivery of pro-inflammatory cytokines, and augment protective enzymes against free radicals [99]. Several clinical studies confirmed that the drug remarkably decreases the employ of parenteral nutrition and the duration of the mucositis, allowing an improvement in the patient’s condition after chemo-radiotherapies [100,101].

## 4. Enhancers of Radiotherapy Activity in Hematologic Neoplasms

As well as the substances capable of carrying out a protective action against the damage caused by RT reported above, other substances have been recognized as possible enhancers of the RT and cytotoxic action (Table 2). The following section summarizes new therapeutic agents that are proposed to be inducers of oxidative stress in hematologic malignancies and may have clinical efficacy in the treatment of B-cell lymphoma and MM.

The appearance of radio-resistance in tumor cells involves a mechanism devised by its intracellular antioxidant system to block the oxidative stress and maintain a low, steady level. It is well recognized that radio-resistance of lymphoma cells accounts for lower basal ROS levels. It is well recognized that GSH concentrations and antioxidant enzymes were greater in lymphoma cells with respect to normal lymphocytes [102].

RT has drawback of toxicity and resistance in cancer cells. A number of natural phytochemicals, such as curcumin, demethoxycurcumin, quercetin, and genistein, are demonstrated to hold radio-sensitizing potential in tumor cells [119,120,121].

Substances that present affinity for electrons can strengthen the biologic action of ionizing radiation [122]. Strengthening may indirectly happen by modifying concentrations of radioprotective metabolites such as glutathione, or the sensitizer may directly cooperate with cellular elements to provoke or increase cellular injury. Models of radiation sensitizers supposed to operate by the former mechanism comprise diamide (diazenedicarboxylic acid *bis*[*N*, *N*_-dimethylamide]), *tert*-butyl hydroperoxide, L-buthionine-(S,R)-sulfoximine (BSO), and other thiol depleters [123,124,125]. Furthermore, nitroimidazoles, tirapazamine, and oxygen directly act with DNA or other targets such as lipid or proteins [126,127]. Generally, oxygen mimetic sensitizers of this latter group are most efficacious under hypoxic situations and the activity is not covered by the competitive action of oxygen. Solvated electrons generated by the radiolysis of water may help to decrease the electron-affinic sensitizer. Nevertheless, even in the absence of ionizing radiation, electron transfer to a radiation sensitizer may happen in the presence of cellular elements that have a more negative standard reduction potential, such as NADPH, flavins, ascorbate, or reduced glutathione [128,129]. This mechanism decreases the reducing substances, which must then be restocked. Moreover, in the presence of oxygen, additional electron transfer can generate reduced oxygen species and restore the sensitizer. Generally, such redox cycling can provoke the onset of a situation of oxidative stress.

**Figure 2:**
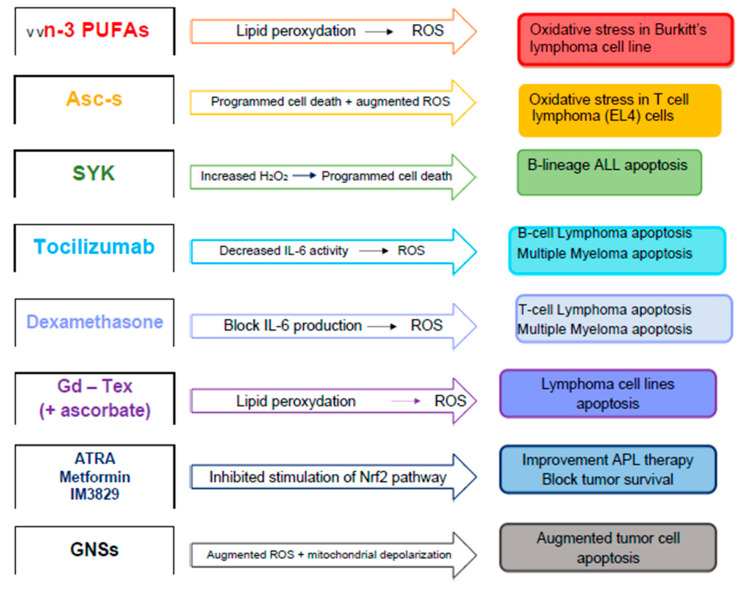
Reactive oxygen species (ROS)-sensitive molecular pathways implicated in survival signaling.

Long-chain n-3 polyunsaturated fatty acids (n-3 PUFAs), such as docosahexaenoic acid (DHA) or eicosapentaenoic acid (EPA), are easily oxidized, and n-3 PUFA-derived peroxidation products are believed essential to justify the cytotoxicity toward tumor cells [130,131,132]. Moreover, their peroxidation may trigger cells to ROS, causing oxidative stress [133]. Additionally, DHA- and EPA-caused oxidative stress may control ROS-sensitive molecular pathways implicated in survival signaling (Figure 2). ROS-sensitive mitogen-activated protein kinases (MAPKs), MAPK-phosphatases [134], and transcription factors [135,136] are essential elements of these pathways. Currently, however, discussion occurs if n-3 PUFAs might also exercise antioxidant actions and decrease ROS production. The paradoxical antioxidant action generated by n-3 PUFAs in several experimental patterns has been justified essentially on the basis of the stimulation of cellular antioxidant elements [103,104,137].

An increased cytotoxic action of irradiation was reported in tumor cells cultured in vitro and treated with DHA and EPA [138]. DHA was also described to block *γ* -radiation-provoked activation of NF-*κ*B in Ramos cells, an extremely radiation-resistant Burkitt’s lymphoma cell line, and to sensitize the cells to radiation-caused programmed cell death [139].

Encouraging results have been also reported in in vivo research in non-hematologic malignancies [105,106,140,141]. This effect was correlated to the DHA-provoked oxidative stress in tumor cells previously subjected to augmented radiation-caused production of ROS.

Ascorbyl stearate (Asc-s) is a byproduct of ascorbic acid with superior anti-tumor effectiveness with respect to ascorbic acid. In a report, authors have evaluated radio-sensitizing action of Asc-s in T cell lymphoma (EL4) cells. Asc-s and radiation administration decreased cell growth, causing programmed cell death in a dose-dependent manner by blocking the cells at S/G2-M phase of the cell cycle [142]. It also reduced the occurrence of tumor stem cells per se, with considerably greater reduction in combination with radiation therapy. Moreover, Asc-s and radiation treatment augmented ROS concentration, a drop in mitochondrial membrane potential (MMP), and augmented caspase-3 activity, thus provoking programmed cell death of EL4 cells. Furthermore, it also drastically reduced GSH/GSSG ratio due to binding of Asc-s with thiols. The augmented oxidative stress caused by Asc-s and radiation treatment was abolished by thiol antioxidants in EL4 cells. Remarkably, this redox modulation stimulated relevant augmentation in protein glutathionylation. Asc-s administration caused glutathionylation of p50-NF-kB, IκB kinase (IKK), and mutated p53, thus blocking tumor expansion during oxidative stress.

A new spleen tyrosine kinase (SYK) P-site inhibitor, 1,4-Bis (9-O dihydroquinidinyl) phthalazine/hydroquinidine 1,4-phathalazinediyl diether (C-61), significantly increased H2O2-caused programmed cell death of primary leukemia cells from each of five relapsed B-lineage acute lymphoblastic leukemia (ALL) subjects [143]. An extremely radiation-resistant subclone of the murine B-lineage leukemia cell line BCL-1 was employed to evaluate the in vivo radio-sensitizing actions of C-61. C-61 increased the antileukemia effectiveness of total-body irradiation (TBI) in a study on syngeneic bone marrow transplantation (BMT) at 20%. It is probable that the use of C-61 into the pretransplant TBI protocols of subjects with high-risk B-ALL will aid the overwhelmed radio-chemotherapy resistance of leukemia cells and thus increase survival outcome [143].

IL-6 might also increase resistance to radiotherapy. In fact, IL-6 plays an important role in promoting myeloma cell growth and chemoresistance; increased IL-6 concentrations are associated with aggressive disease and poor outcome [144,145]. IL-6 has been demonstrated to induce superoxide generation in monocytes, neutrophils, and neuronal cells [146,147]. However, surprisingly, IL-6 can provoke adaptive responses to oxidative stress in normal cells by protecting cells from hydrogen peroxide-caused cell death [148]; transgenic animals overexpressing IL-6 demonstrate resistance to hyperoxia [149]. Although MM subjects display augmented lipid peroxidation and lower concentrations of antioxidant enzymes in plasma and erythrocytes [150,151,152,153,154,155,156], research reported the effect of IL-6 in restoring intracellular redox homoeostasis in the setting of myeloma treatment. IL-6 employment augmented myeloma cell resistance to substances that provoke oxidative stress, comprising IR and Dex (dexamethasone) [157]. As compared to IR alone, myeloma cells treated with IL-6 plus IR showed decreased caspase-3 stimulation, annexin/propidium iodide staining, PARP [poly(ADPribose) polymerase] cleavage, and mitochondrial membrane depolarization with augmented clonogenic survival. IL-6 combined with IR or Dex augmented early intracellular pro-oxidant concentrations that were correlated to stimulation of NF-*κ*B. In myeloma cells, upon combination with hydrogen peroxide administration, relative to TNF-*α*, IL-6 caused an alteration of decreased glutathione concentration and augmented NF-*κ*B-dependent manganese superoxide dismutase (MnSOD) expression. Furthermore, knockdown of MnSOD reduced the IL-6-caused myeloma cell resistance to radiation. MitoSOX Red staining demonstrated that IL-6 administration reduced late mitochondrial oxidant generation in irradiated myeloma cells [157]. Moreover, in B-cell lymphoma, radio-resistance has been also associated with the presence of IL-6-producing tumor cells [158].

The use of substances with anti-IL-6 activity (tocilizumab) should be explored in an attempt to increase sensitivity to radiotherapy.

From this point of view, the action of steroids could be interesting. Dexamethasone has been reported to provoke oxidative cell death in T-cell lymphoma [107]; nevertheless, Dex effect in causing oxidative stress in MM has not been proved [108,159].

Bera et al. suggested a new combination of radiation plus Dex that presents greater clonogenic cell killing and programmed cell death of myeloma cells and eradicates myeloma cells when cultured with bone marrow stromal cells (BMSCs) [109]. Dex was reported to block the production of IL-6 from irradiated BMSCs. In 5TGM1 model, combined use of Dex with skeletal radiotherapy (153-Sm-EDTMP) increased median survival time and reduced radiation-caused myelosuppression. Dex augmented superoxide and hydrogen peroxide generation and increased radiation-caused oxidative stress and cell death of myeloma cells. However, Dex reduced radiation-caused augmentation in pro-oxidant concentrations and increased the clonogenic survival in progenitor cells and normal hematopoietic stem cells. Administration with either N-acetylcysteine or the combinate use of polyethylene glycol (PEG)–conjugated copper, PEG-catalase, and zinc-superoxide dismutase considerably safeguarded myeloma cells from Dex-caused clonogenic death [109]. These findings state that Dex in combination with RT increases the destroying of myeloma cells while defending normal bone marrow hematopoiesis via a system that implicates an augmentation in oxidative stress.

Magda et al. evaluated the mechanism of radiation improvement by motexafin gadolinium (Gd-Tex) in vitro [110]. Clonogenic assays were employed to evaluate radiation response in lymphoma cell lines. Gd-Tex catalyzed the oxidation of NADPH and ascorbate under aerobic conditions, generating hydrogen peroxide. Cultivation with Gd-Tex in the presence of ascorbate augmented the aerobic radiation response of E89. Gd-Tex sensitizes cells to IR by augmenting oxidative stress [110].

Finally, RT, chemotherapy, and immunotherapy are the main treatments in Non-Hodgkin’s lymphoma therapy. There is the probability that the combination of chemotherapy or immunotherapy and RT may have a synergistic effect. In one report, rituximab was reported to radio-sensitize lymphoma cells controlling programmed cell death and cell cycle-related proteins [111,112,113,160].

The stimulation of apoptosis caused by the combined use of RT and rituximab was demonstrated to be caused by mitochondrial dissipation. ROS were described to have a role in this phenomenon [161]. Fengling et al. evaluated the action of rituximab on cell death caused by radiation in Raji lymphoma cells [114]. G2/M cell cycle arrest was reported after irradiation alone and the combination therapy. The combination therapy caused an increase in ROS production in a radiation dose-dependent manner. Moreover, rituximab increased the cell inhibition and programmed cell death caused by H2O2 [114].

An interesting field of study is the Kelch-like ECH-associated protein 1(Keap1)-nuclear factor erythroid 2-related factor 2 (Nrf2). It is the most investigated signaling pathway of cellular defense against oxidative stress [115]. When ROS concentrations are augmented, Nrf2 detaches from its inhibitor, Keap1, and translocates into the nucleus where it creates a heterodimer with small V-Maf Avian Musculoaponeurotic Fibrosarcoma Oncogene MAF proteins (sMAF) and controls the expression of genes that comprise the antioxidant response elements. Nrf2 regulates about 250 genes, essentially implicated in the endogenous antioxidant defense and detoxification of ROS. Mutation of Nrf2, or of its negative regulator Keap1, can alter their relations, which may explain the overexpression of Nrf2 signaling [115]. While normal cells are defended from DNA injury caused by ROS, tumor cells are protected against chemo- or radiotherapy [115].

Nevertheless, Nrf2 stimulation is not general. For instance, it was observed that after RT, the majority of Nrf2-targeted genes remained unchanged. Moreover, there were nine genes implicated in lipid peroxidation, which showed under expression in the case of new RT [116]. Recently, new data suggest that Nrf2 has conflicting actions in tumors. Anomalous stimulation of Nrf2 is correlated with poor prognosis as lasting stimulation of Nrf2 considerably increases the tumor cell resistance to ROS by increasing antioxidant enzymes. The increased Nrf2 in RT is recognized to be correlated with greater expression of Nrf2 downstream targets that stimulate GSH synthesis. Affecting Nrf2 activity in hematologic malignancies and tumors may be an efficacious approach to reduce radioresistance [116] and several Nrf2 inhibitors detected. For instance, All-trans-retinoic acid, employed for the therapy of acute promyelocytic leukemia, has been suggested as a specific Nrf2 inhibitor, which permits Nrf2 to form a complex with retinoid X receptor alpha (RARα), inhibiting stimulation of the Nrf2 pathway [117]. Moreover, metformin, a drug commonly used in the therapy of type 2 diabetes, decreases mRNA and protein concentrations of Nrf2 via the block of Raf/ERK/Nrf2 signaling [118], while IM3829 (4-[2-Cyclohexylethoxy] aniline) reduces Nrf2 mRNA and protein concentrations, and combination with radiation is capable to considerably block tumor survival [162].

Moreover, other radiosensitizers, which can considerably increase the radiotherapeutic efficacy, have been produced. Gold-based nanomaterials, as a novel sort of nanoparticle-based radiosensitizer, have been employed in experimentations implicating tumor RT [163]. However, therapeutic action employing the gold nanoparticle-based RT is generally not significant because of the low cellular uptake effectiveness and the autophagy-inducing capacity of these gold nanomaterials. Using gold nanospikes (GNSs), investigators built a series of thiol-poly(ethylene glycol)-modified GNSs terminated with folic acid (FA) (FA-GNSs), amine (NH_2_-GNSs), methoxyl (GNSs), and the cell-penetrating peptide TAT (TAT-GNSs), and studied their actions on X-ray RT [164]. The sensitization enhancement ratio (SER), which is generally employed to assess how efficiently radiosensitizers reduce cell growth, reaches 2.30 for TAT-GNSs. The exceptionally elevated SER value for TAT-GNSs suggests the greater radiosensitization action of this nanomaterial. The radiation improvement system of these GNSs implicated the augmented ROS and mitochondrial depolarization. Surface-modified GNSs could provoke the increase of autophagy-related protein (LC3-II) and apoptosis-related protein (active caspase-3) in tumor cells. GNSs provoked the diminishing of autolysosome degradation ability and autophagosome amassing. These findings established that autophagy has a protecting action against RT, and the block of defensive autophagy with inhibitors would provoke an augmentation of cell apoptosis. As well as the above in vitro experiments, the in vivo studies also reported that X-ray + TAT-GNSs therapy had the best tumor proliferation inhibitory action, which proved the greatest radiation sensitizing action of TAT-GNSs [164].

Finally, some drugs that have long been used in the treatment of haematological neoplasms also seem to have radiosensitizing capabilities. For instance, fludarabine is also a well-known DNA damage repair inhibitor that has clinical activity against hematological cancers [165].

In an in vitro experimental study, fludarabine-P, in clinically possible dosages, is a potent radiosensitizer in a human squamous carcinoma cell line of the oropharynx (ZMK-1 cells) and has a minor action in the fetal lung fibroblasts (MRC-5) [166]. The radiosensitization of fludarabine-P appears to be over additive in the malignant cells and additive in normal fetal fibroblasts. This would suggest that fludarabine-P might increase the therapeutic ratio of radiation.

Another well-known radiosensitizer is hydroxyurea, which was used in treatment of hematological malignancies [167], and several studies demonstrated that the addition of hydroxyurea to ionizing radiation produced a synergistic effect in vitro [75,168].

## 5. Future Perspectives

RT is a keystone of both curative and palliative tumor treatment. However, RT is harshly limited by radiation-caused side effects. If these side effects could be decreased, a bigger dosage of radiation could be given to obtain a superior response. Pre-clinical experimentations have demonstrated that irradiation at dosage rates far exceeding those generally employed decreases radiation-induced side-effects though conserving a comparable anti-tumor effect. This is recognized as the FLASH effect [76]. To date, numerous subjects have been treated with Ultra-High Dose Rate (FLASH) RT for the therapy of lymphomas causing complete responses and reduced toxicities. The mechanism accountable for decreased tissue toxicity is yet to be clarified, but the most noticeable theory so far suggested is that acute oxygen diminution happens within the irradiated tissue [76].

In the near future, other areas of intervention will open up in the field of radiation protection, such as that of non-coding genetic material. Previous reports have recognized essential actions of specific miRNAs in radiation response in numerous malignancies [169].

miR-139-5p is a powerful controller of RT response in tumors through its modulation of genes implicated in several DNA repair and ROS protection pathways. Therapy of solid cancer cells with a miR-139-5p mimic intensely synergized with radiation both in vitro and in vivo, causing a relevant augmentation of oxidative stress and stimulation of programmed cell death. Numerous miR-139-5p target genes were also predictive of prognosis. These prognostically important miR-139-5p target genes were employed as markers to recognize radioresistant cancer xenografts extremely responsive to sensitization by treatment with a miR-139-5p mimetic [170]; one of its confirmed targets, MAT2A, has a role in ROS defense [171].

The use of specific antisense oligonucleotides, able to bind and antagonize miRNAs, could be efficacious as a therapeutic element able of provoking a diverse response to oxidative stress and RT [172].

A different field of study could be the neuroprotection. Primary Central Nervous System Lymphoma (PCNSL) is a non-Hodgkin lymphoma that occurs within the brain, spinal cord, or eyes in the absence of systemic disease. Therapy often comprises whole-brain radiotherapy. However, after therapy, some subjects may show neurologic alterations [173]. This delayed treatment-related side-effect appears to be similar to a diffuse leukoencephalopathy. Some data indicate that the mechanism is related to the disruption of frontal subcortical circuits provoked by radiation injury, probably produced by oxidative stress [174].

Platelet-rich plasma (PRP) is an autologous preparation of platelets which comprehends great amounts of growth factors such as platelet-derived growth factor, epidermal growth factor, vascular endothelial growth factor, hepatocyte growth factor, insulin-like growth factor-1, and transforming growth factor-beta 1 [175]. PRP pre-treatment considerably decreased the radiation-induced alterations. Moreover, PRP remarkably improved the state of oxidative stress and seemed to block apoptotic process [176]. A similar approach could be attempted to prevent RT-induced neurological toxicity.

In the future, the possibility of modifying the radiosensitivity of the different cell types could be decisive in guaranteeing the effectiveness of the radiotherapy treatment. The L5178Y lymphoma cells (LY-S cell line) were the first ionizing radiation-sensitive mammalian cell line to be described. Investigators isolated the LY-S line from the parental L5178Y line (later called LY-R; R for resistant) and studied its high radiosensitivity. The following studies were targeted at comprehending the cause for the radiation sensitivity difference between the LY lines [177]. The LY-R to LY-S phenotype modification was due to the oxidative shock after cell relocation from the ascitic tumor into culture medium [177].

Full knowledge of the intergenomic interactions seems crucial for understanding the cellular response to ionizing radiation. Finding therapeutic solutions for hematologic malignancies and other diseases may also depend upon such knowledge.

## 6. Conclusions

Ionizing radiation has a main function in modern tumor treatment due to its distinctive advantages comprising non-invasiveness and a lack of severe systemic toxicity. Even though RT has showed several degrees of success, relapse and treatment failure may happen in tumor subjects due to radioresistance. Therefore, approaches are urgently necessitated for increasing radiosensitivity of tumor cells and augmenting the radioprotection of normal cells. The elaboration of novel and clinically efficacious modulators will offer prospects for new treatment paradigms.

In this compound, planning complementary therapeutic methods founded on differences in oxidative metabolism between tumoral and normal cells could be an effective approach to create more successful treatments.

## Figures and Tables

**Table 1:** Radioprotective agents able to reduce the harmful effects of the treatment.

Agent	Mechanisms of Action	Limits	Advantages	Ref.
Phenolic (flavonoids, stilbenes, tannins, lignans, lignins, quercetin, genistein)	Inhibition of apoptosis	Limited water solubility and poor availability		[49,50,51]
Polyphenolic—polysaccharide conjugates	Inhibition of apoptosis		Non-toxic, water-soluble polymeric compounds. They did not protect the leukemic cells against radiation-induced apoptotic death.	[52,53]
Cimetidine	Hydroxyl radical scavenging mechanism. Decrease of inflammation, and Bax/Bcl2 ratio.		Reduction of loss of bone marrow cell count, intestinal lining destruction, and fibrosis. Differential effect on both cancer cells and adjacent healthy cells.	[54,55]
Amifostine (WR2721)	Organ repair via bone marrow recruitment or dedifferentiation.	Intolerance and significant accumulative toxicity.		[56]
STW5 (Iberogast)	Anti-apoptotic effects.	Antioxidant activity and anti-inflammatory properties	Action on radiation enteritis. Preservation of the mucosal integrity of the small intestine.	[57,58,59,60]
CBLB502	Increased expression of the strong natural antioxidant superoxide dismutase and induction of radioprotective cytokines (G-CSF, IL-6, and TNF-α). Suppression of p53-dependent apoptosis. Reduction of DNA damage and chromosomal aberrations. Action on the TLR5 signaling pathway.		It protects mammals from gastrointestinal and hematopoietic acute radiation syndrome. Reduction of IR-induced oxidative stress, reduction of decline of sperm quantity and quality.	[61,62,63,64,65] [66,67]
Erdosteine	Protective role on the release of free oxygen radicals. Action on TNF alpha, interleukin 1, and IL-6.		Protection against radiation induced inflammatory kidney damage.	[68]
Human umbilical cord-derived mesenchymal stromal cells	Prevention of oxidative stress and increased antioxidant status. Reduction of pro-fibrotic TGF-β1, IL-6, and IL-8 levels.		Protective effects on irradiation myocardial fibrosis with increased cell viability, reduction of collagen deposition.	[69]
Calf spleen extractive injection	Regenerating action on damaged cells.		Reduction of thrombocytopenia and leucopenia.	[70,71,72]
Polyxydroxylated fullerenes	Anti-oxidative effects.		Prevention of radiation-induced reduction in the white cell count.	[73,74]
Platelet-rich plasma	Administration of growth factors. Reduction of oxidative stress and inhibition of the induced apoptosis.		Neuroprotection.	[75,76]

**Table 2:** Enhancers of radiotherapy activity in hematologic neoplasms.

Agent	Mechanisms of Action	Effects	Ref.
Natural phytochemicals (curcumin, demethoxycurcumin, quercetin, genistein)	Alteration of levels of radioprotective metabolites. Electron transfer to a radiation sensitizer.	Reduction of radio-resistance.	[100,101,102]
Long-chain n-3 polyunsaturated fatty acids	Their peroxidation may sensitize cells to ROS, inducing an oxidative stress. Modulation of ROS-sensitive mitogen-activated protein kinases and phosphatases, and transcription factors.	Cytotoxicity. Increased radiation-induced apoptosis.	[103,104]
Ascorbyl stearate	Augmented levels of ROS, drop in mitochondrial membrane potential and increased caspase-3 activity.	Reduction of cell proliferation, induction of apoptosis by arresting the cells at S/G2-M phase of cell cycle.	[105]
Spleen tyrosine kinase (SYK) P-site inhibitor	Increased H2O2-induced apoptosis.	Action on radio-chemotherapy resistance.	[106]
Dexamethasone	Increased superoxide and hydrogen peroxide production and augmented radiation-induced oxidative stress.	Clonogenic cell killing and apoptosis of myeloma cells.	[107]
Rituximab	Elevation in ROS generation	Increase of cell growth inhibition. Augmented apoptosis.	[108,109,110,111,112,113]
All-*trans*-retinoic acid, Metformin, IM3829	Inhibition of nuclear factor erythroid 2-related factor 2	Inhibition of cancer cell survival.	[114,115,116]
Gold nanoparticle-based compounds	Increased ROS levels, mitochondrial depolarization, and cell cycle redistribution.	Inhibition of protective autophagy.	[117,118]
